# The majority of total nuclear-encoded non-ribosomal RNA in a human cell is 'dark matter' un-annotated RNA

**DOI:** 10.1186/1741-7007-8-149

**Published:** 2010-12-21

**Authors:** Philipp Kapranov, Georges St Laurent, Tal Raz, Fatih Ozsolak, C Patrick Reynolds, Poul HB Sorensen, Gregory Reaman, Patrice Milos, Robert J Arceci, John F Thompson, Timothy J Triche

**Affiliations:** 1 Helicos BioSciences Corporation, One Kendall Square, Building 700, Cambridge, MA 02139, USA; 2 Department of Molecular Biology, Cell Biology and Biochemistry, Brown University, SFH Life Sciences Building, 185 Meeting St, Providence, RI 02912, USA; 3 Cancer Center, Departments of Cell Biology & Biochemistry, Pediatrics, and Internal Medicine, School of Medicine, Texas Tech University Health Sciences Center, 3601 4th Street STOP 9445, Lubbock, TX 79430-6450, USA; 4 British Columbia Cancer Research Centre, 675 West 10th Avenue, Room 4112, Vancouver, BC, Canada V5Z 1L3; 5 Department of Pediatrics, The George Washington University School of Medicine and Health Sciences, Division of Oncology, Children's National Medical Center, 11 Michigan Ave, NW, Washington, DC, 20422, USA; 6 Kimmel Comprehensive Cancer Center at John Hopkins, Department of Oncology/Pediatric Oncology, The Buntings Blaustein Cancer Research Building, 1650 Orleans Street, Suite 207, Baltimore, MD, 21287, USA; 7 Department of Pathology, University of Southern California, 1975 Zonal Avenue, Los Angeles, CA 90089-9034, USA; 8 Grupo de Inmunovirologia, SIU, Universidad de Antioquia, Calle 67 Número 53 - 108, Medellin, Antioquia, Colombia

## Abstract

**Background:**

Discovery that the transcriptional output of the human genome is far more complex than predicted by the current set of protein-coding annotations and that most RNAs produced do not appear to encode proteins has transformed our understanding of genome complexity and suggests new paradigms of genome regulation. However, the fraction of all cellular RNA whose function we do not understand and the fraction of the genome that is utilized to produce that RNA remain controversial. This is not simply a bookkeeping issue because the degree to which this un-annotated transcription is present has important implications with respect to its biologic function and to the general architecture of genome regulation. For example, efforts to elucidate how non-coding RNAs (ncRNAs) regulate genome function will be compromised if that class of RNAs is dismissed as simply 'transcriptional noise'.

**Results:**

We show that the relative mass of RNA whose function and/or structure we do not understand (the so called 'dark matter' RNAs), as a proportion of all non-ribosomal, non-mitochondrial human RNA (mt-RNA), can be greater than that of protein-encoding transcripts. This observation is obscured in studies that focus only on polyA-selected RNA, a method that enriches for protein coding RNAs and at the same time discards the vast majority of RNA prior to analysis. We further show the presence of a large number of very long, abundantly-transcribed regions (100's of kb) in intergenic space and further show that expression of these regions is associated with neoplastic transformation. These overlap some regions found previously in normal human embryonic tissues and raises an interesting hypothesis as to the function of these ncRNAs in both early development and neoplastic transformation.

**Conclusions:**

We conclude that 'dark matter' RNA can constitute the majority of non-ribosomal, non-mitochondrial-RNA and a significant fraction arises from numerous very long, intergenic transcribed regions that could be involved in neoplastic transformation.

## Background

A variety of techniques, most notably tiling arrays [1-4], massive sequencing of complimentary DNAs (cDNAs) [5,6] and cDNA tags [7], has consistently identified the presence of RNA molecules encoded from regions of the genome not currently annotated as exons of protein-coding RNAs in human cells [8]. Collectively, these RNA molecules were dubbed 'dark matter in the genome' [9]. While these approaches underscored the complexity of transcriptional output from the human genome [10], tiling arrays could predict only the complexity, but not the relative mass of 'dark matter' RNA as a fraction of total transcription and, hence, they could not make strong conclusions about its importance. Similarly, the majority of methods for cDNA sequencing have either used polyA+ selected RNA and/or amplification for library construction, both of which selectively omit significant amounts of RNA and, hence, cannot accurately reflect the profile of all cellular RNAs. These technological limitations in assessing all cellular RNAs have led to the notion that non-coding RNAs (nc-RNA) represent only a minor proportion of the total mass of RNA molecules relative to protein-coding species and may arise only as a by-product of transcriptional noise [9,11]. If so, they would not be worthy of additional analysis while others have suggested that the situation merits further study [12].

The advent of next-generation sequencing technologies and their application to RNA analysis [13] provides the opportunity for us to re-assess the relative mass of the nc-RNAs and, thus, directly pose the question of how prevalent and important this class of RNAs might be. However, care must be taken to ensure the RNA analysed is representative of the entire cell and that the limitations of the technologies being used are understood to minimize the impact of any biases. One initial report [14] has suggested that nc-RNAs represent only a minor fraction of RNA in a mammalian cell and that most of these non-coding species consisted of intronic by-products generated from unspliced RNAs. In order to further address this question, we have used single-molecule sequencing (SMS) to reduce the potential for representational biases by avoiding amplification and minimizing sample preparation [15,16]. We show that when total RNA is sequenced rather than just the highly selected polyadenylated RNAs, 'dark matter' RNAs represent a significant fraction, sometimes even the majority, of the stable non-ribosomal, non-mitochondrial RNAs (mtRNA) in a cell. Furthermore, gene-desert regions, currently devoid of annotations, are found to be expressed at abundant levels and RNA species from these regions may be over-represented during or following neoplastic transformation.

We provide estimates of the relative mass of the 'dark matter' RNA in cells of two organisms - human and fly. To our knowledge, while used in a number of reports, this term has not been defined and, thus, we offer our definition of this term: 'dark matter' RNA includes any RNA whose function we do not currently understand and any RNA that is currently un-annotated. The latter category may include both protein-coding and nc-RNAs. A more detailed breakdown of what is included and how these species might arise is provided in the Additional File 1: Definition of dark matter.

Another parameter requiring definition is the measurement for transcriptome complexity used here. In general, the fraction of 'dark matter' RNA could be measured either as a fraction of nucleotide bases in the genome that are used to encode these transcripts or as the relative mass of these RNAs as a fraction of mass of all RNAs in the cell after removing ribosomal and mtRNAs. Most techniques, notably tiling arrays have provided the former estimate. Importantly, however, this estimator alone cannot provide a complete description of the complexity of 'dark matter' RNA as it is possible that it can be highly complex in terms of the sequences present in this population and yet comprise a minute fraction of total RNA population by mass. This has been suggested to be the case by the recent report of van Bakel et al. who estimate that 'dark matter' RNA constitutes 12% of all RNA by mass [14]. In that report, the authors have leveraged the ability of a next-generation sequencing to count individual sequencing reads and assign them to either known or 'dark matter' RNA categories. The fraction of total reads in each category of RNA represents its relative mass. We have also used the relative mass as defined by fraction of reads falling within a certain category as an estimator of the abundance of RNA. We used the number of reads as opposed to the total number of nucleotides covered by the reads to estimate the relative mass of RNA since the SMS reads do not have the same lengths.

## Results and discussion

**Figure 1:**
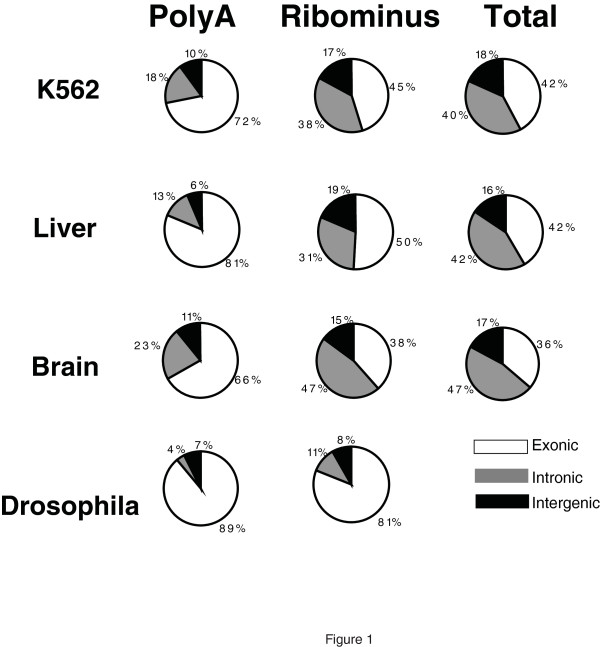
**Distribution of single-molecule sequencing (SMS) reads among exonic, intronic and intergenic regions in polyA+, RiboMinus and total RNA**. RNA samples were prepared as described in the Materals and Methods section of the paper for sequencing. Each source of RNA [K562 cells, human liver or brain tissue and adult flies (*Drosophila*)] was used either directly (total RNA) or after fractionation by RiboMinus treatment or selection for the polyA containing RNAs. Each sample was sequenced on one or more channels. Reads were aligned to hg18 or dm3 version of the human or fly genomes. After the removal of reads that aligned to the mitochondrial and ribosomal sequences, the remaining sequences were assigned as exonic (white), intronic (grey) or intergenic (black) based on the University of California Santa Cruz genes database and the percentages found for each are shown as pie charts. The exact read data can be found in Additional File 2: Table S1.

In order to evaluate the true nature of the complete cellular RNA population, we analysed RNA from various tissue sources and different RNA preparations in two different species: human and Drosophila (Figure 1 and Additional File 2: Table S1). The filtered sequence reads used in this work have been deposited in the National Center for Biotechnology Information Short Read Archive [SRA:SRP004776]. PolyA+ RNA, total RNA and total RNA depleted of ribosomal RNA were studied. As described in the Materials and Methods section, RNA preparations were treated with saturating concentrations of DNAse I followed by purification over columns to eliminate small amounts of any remaining partially-digested small DNA pieces. This step can also remove a substantial fraction of RNAs below 200 bases and, thus, this work is primarily aimed at RNAs longer than 200 bases. The priming of RNA with random hexamers and reverse transcription generated first-strand cDNA which was then tailed with terminal transferase and deoxyadenosine triphosphate (dATP). This was followed by sequencing using single-molecule sequencing after hybridization to a flow cell surface with covalently bound poly dT50 [17]. No amplification, ligation or size selection were used in cDNA preparation, minimizing methodological biases [18]. Each cDNA sample was sequenced on one or more channels of a HeliScope Genetic Analysis System with the resulting filtered reads (25-55 bases, average read length 33-36 bases depending on the run) aligned to the complete human or Drosophila genomes supplemented with the sequence of the complete ribosomal DNA (rDNA) repeat (Materials and Methods section). Reverse transcription is known to produce spurious second-strand cDNA [19], so reads from both strands of the genome were combined in order to avoid sense/antisense artifacts. The unique mapping reads from human tissue sources were further filtered to exclude sequences aligned to rDNA sequences, the mitochondrial genome, as well as to genomic repeats annotated by the RepeatMasker program as rRNA. After filtering, the remaining informative reads were used for subsequent analyses, including a comparison to the known annotations defined by the University of California, Santa Cruz (UCSC) Genes or the FlyBase Genes tracks from the UCSC browser.

As shown in Figure 1 and Additional File 2: Table S1, the highest fraction of non-exonic reads consistently occurred in total RNA (approximately 51%-64%) and total RNA depleted of ribosomal RNA (RiboMinus RNA, approximately 50%-62%). Strikingly lower proportions of non-exonic reads (or ncRNAs) occurred in the polyA+ fraction, although they still accounted for approximately 19%-33% of the informative reads. Two different polyA+ RNA preparation methods for human liver RNA, using either magnetic beads or oligo-dT cellulose purification, produced similar yields of informative reads (81% and 84.4%, respectively) that overlap known exons. Tissue source had a modest effect on the fraction of non-exonic RNAs, with the normal brain having the highest proportion of ncRNA transcripts, followed by the leukaemia cell line K562 and then the liver, which showed the lowest level. Since these data were not biased by potential effects of amplification or ligation, they are more likely to accurately represent the relative mass of different populations of RNAs than other techniques which require additional processing steps. Any skewing in favour of non-exonic RNAs that could be induced by the process of RNA purification from the cell or reverse transcription would have also occurred to a similar extent in other studies. Overall, this suggests that the un-annotated transcriptome, as defined by the reads mapping outside exons of protein-coding RNAs, represents the majority of RNAs by mass, comprising up to two-thirds of all non-ribosomal, non-mtRNA in a human cell. However, of interest and consistent with the previous observation based on tiling arrays [20], RNA from adult *Drosophila *flies did not have as much non-exonic RNAs by mass as human cells, comprising approximately 11% of the polyA+ RNA and 19% of the RiboMinus RNA. This is consistent with earlier observations based on tiling arrays suggesting that *Drosophila *does not have as much 'dark matter' RNA as human cells [20] and supports the previous suggestion that the fraction of the 'dark matter' RNA increases with increasing organismal complexity [21]. As with the human samples, the fly samples have a higher fraction of 'dark matter' RNA in total rather than polyA+ RNA (Figure 1 and Additional File 2: Table S1). The data and analysis below refer to human samples unless otherwise specified.

**Figure 2:**
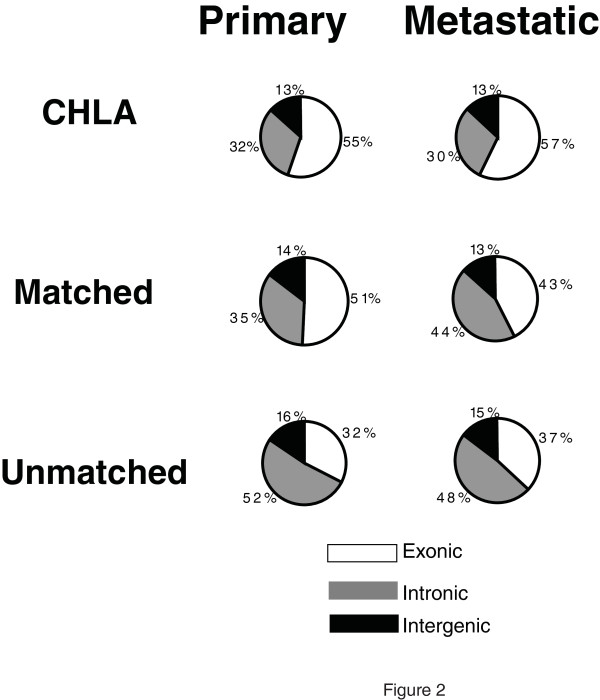
**Distribution of single-molecule sequencing (SMS) reads among exonic, intronic and intergenic regions in RiboMinus RNA of different Ewing Family of Tumours samples**. RNA samples were prepared as described in the Materials and Methods section of the paper for sequencing. Each source of RNA (from immortalized cell lines (CHLA), from primary and metastatic tumours, from primary and metastatic tumours from one individual (matched) and from primary and metastatic tumours from different individuals (unmatched), was used after the removal of most of the ribosomal RNA by RiboMinus treatment. Each sample was sequenced on one or more channels. Reads were aligned to the hg18 or dm3 version of the human or fly genomes. After removal of reads that aligned to mitochondrial and ribosomal sequences, the remaining sequences were assigned as exonic (white), intronic (grey) or intergenic (black) based on the University of California Santa Cruz genes database and the percentages found for each are shown as pie charts. The exact read data can be found in Additional File 3: Table S2

In order to further investigate the complexity of transcriptional output in human samples, we have analysed six different Ewing Family of Tumours (EFT) samples from four different patients. These included a pair of matching cell lines derived from a single patient, one from the primary-tumour established in culture prior to chemotherapy (CHLA-9) and one from a metastatic-lesion cultured after chemotherapy (CHLA-10). A second matched set of samples was derived from primary and metastatic EFT tumours from a different patient. An additional primary and metastatic tumour sample were each obtained from different patients (see Materials and Methods section). The primary and metastatic tumour samples served to exclude the possibility that the observed non-exonic RNAs are an artifact of tumour cell line culture conditions. Figure 2 and Additional File 3: Table S2 summarizes the analysis of these samples profiled using RiboMinus RNA. Consistent with the previous results, non-exonic RNAs represented 43%-63% of all non-ribosomal, non-mtRNAs by mass. Primary tumour samples had more non-exonic RNAs (50%-63%) than the cell lines (43%-45%), which could be due to the more heterogeneous nature of the tumour tissues. Tumours are known to be comprised of multiple cell types with normal tissue stroma yielding up to 50% of the total RNA mass and different cell-types could have different non-exonic RNAs expression profiles [2].

**Figure 3:**
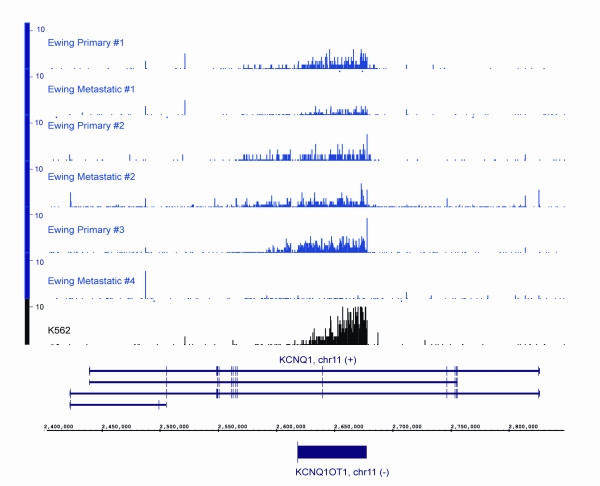
**Detection of a known intronic non-coding RNA (ncRNA) *KCNQOT1 *in RiboMinus RNAs from Ewing Family of Tumours (EFT) and K562 tumour samples**. Gene expression arising from chromosome 11, positions 2,400,000 to 2,800,000 (near the *KCNQ1 *gene) is shown for seven different RNA samples, six EFT samples and the K562 cell line. For each sample, the Y axis (0-10) shows the density of reads per genomic base overlapped by at least one read in 10 million non-ribosomal, non-mitochondrial reads with the X-axis showing the chromosomal position. The location of annotated exons on the sense strand (+) for *KCNQ1 *is shown between the chromosomal position and the expression levels for each sample. The position of the antisense (-) intronic ncRNA *KCNQ1OT1 *is shown below the chromosomal position. All gene annotations and genomic coordinates are based on University of California Santa Cruz genes and hg18 version of the genome.

**Figure 4:**
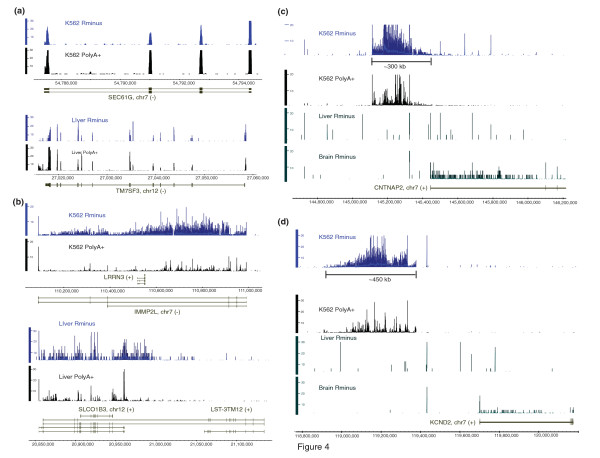
**The presence of abundant non-exonic RNAs in introns and intergenic regions in human cells**. Chromosomal locations and gene regions that display different patterns of intronic or intergenic expression are shown in panels A-D. For each region, the chromosome, annotated gene and strand are shown at the bottom with annotated exons represented by boxes. Above the exons, chromosomal positions based on the University of California Santa Cruz genes and the hg18 version of the genome are shown. The source of the sample RNA (K562, liver or brain) and the type of RNA preparation (RiboMinus or polyA selected) are shown next to the Y-axis. Examples of loci producing little or large amounts of intronic RNAs are shown in panels A and B. Examples of very long intergenic transcribed regions are shown in panels C and D. The Y axis show the density of reads per each genomic base overlapped by at least one read in reads per 10 million of non-ribosomal, non-mitochondrial reads.

Thus, overall, 30%-52% of all informative reads in the human RiboMinus RNA did not overlap exons and fell within intronic regions (Additional File 2: Table S1, Additional File 3: Table S2, Figures 1 and 2) with an average of 40%, representing a significantly higher fraction than the 5.8% reported by van Bakel *et al. *[14]. Intronic reads could simply represent unspliced RNAs and to some extent this will be the case. However, the abundance and specificity that we find among intronic RNAs indicate they are not simply bystanders but suggest a more complex role. Introns have been shown in several instances to harbour known non-coding functional RNAs, such as the *KCNQ1OT1 *transcript involved in imprinting [22]. By RNA mass in a human cell, transcripts emanating from intronic sequences approximately equal that of exonic sequences but this large amount of intronic sequence cannot be explained just by the fact that introns are longer and, thus, accumulate more reads. The density of reads from individual introns can be quite abundant and similar to, or higher than, that of exonic regions. This is exemplified by the known ncRNA *KCNQ1OT1 *embedded within the protein-coding *KCNQ1 *locus and transcribed from the opposite strand, indicating it is not simply a splicing artifact (Figure 3). Additional examples in loci not currently known to harbour ncRNAs are shown on Figure 4b. In contrast, RNA transcripts from some introns are virtually absent (see Figure 4a). In the *IMMP2L *and *SLCO1B3/LST-3TM12 *loci (Figure 4b), the density of reads in intronic regions is equal to, or higher than, in the exonic regions, with a notable enrichment of intronic sequences in the RiboMinus compared to polyA+ RNA. Overall, such examples are not rare. If we consider all spliced UCSC Genes annotations expressed in K562 defined by the presence of at least 50 reads per 10 M in intronic and exonic regions, we identify 34,962 transcripts. Of these, 3760 have at least one intron whose average read density is equal to, or higher than, the density of the exons. It is also important to emphasize that we obtained these results from an analysis of cellular total RNA, not nuclear-enriched RNAs and, thus, the relative mass of introns in the total RNA pool is impressive.

Overall, our results are consistent with the fact that introns produce abundant stand-alone transcripts. Consistent with this, Louro *et al. *[23] have found that over 80% of human RefSeq loci have 78,147 transcriptional units formed by clusters of expressed sequence tags (ESTs) that map totally within introns, suggesting that introns contain transcripts that are not part of pre-messenger RNA. Importantly, these units are not randomly distributed in the genome [24]. Furthermore, intronic RNAs were previously found to represent a compilation of different RNA species in addition to independent sense and antisense transcripts - transcripts retaining specific introns as well as RNA molecules with alternative exons and alternative un-translated regions [4].

Although less frequent than intronic reads, a substantial number of RiboMinus RNA reads also mapped to intergenic space. Intergenic reads comprise 24%-37% of all non-exonic reads and approximately 15%-18% of all informative reads in the RiboMinus RNA samples (Figure 1 and Additional File 2: Table S1). Interestingly, we also noticed a number of very long, abundant intergenic transcribed regions spanning hundreds of kbs of genome virtually devoid of annotations, as illustrated in Figures 4c and 4d. Similar to the intronic RNAs, these transcripts were also enriched in the RiboMinus RNAs, although still detectable in polyA+ RNA samples.

**Figure 5:**
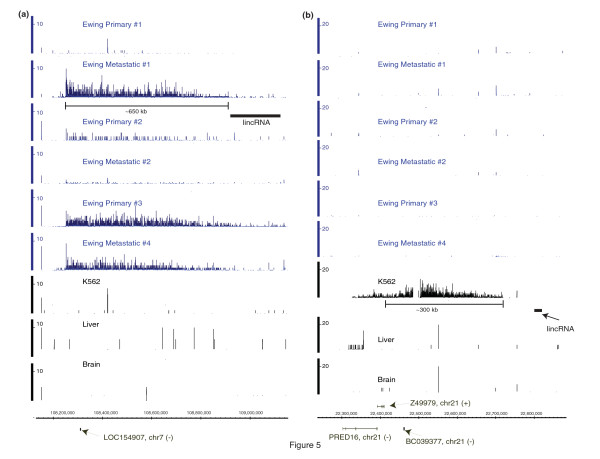
**An example of very long transcribed intergenic regions identified in tumour cells**. An example of a locus with high expression between annotated genes on chromosome 7 that was found in several Ewing Family of Tumours (EFT) cell lines and tissues but not in K562 or normal tissues is shown in panel A with chromosomal position along the X axis. A locus on chromosome 21 that was found to have high expression in K562 but not EFT samples or normal tissues is shown in panel B. The EFT primary No,1 and metastatic No.1 samples correspond to the CHLA-9 and CHLA-10 cell lines (see the Materials and Methods Section of the paper) and the remainder are from patient EFT samples, K562 cells, or normal tissues (liver and brain). The K4-K36 domains which harbour large intergenic non-coding RNAs, as reported by Khalil *et al. *[27], are also shown. The Y axis show the density of reads per each genomic base in 10 million non-ribosomal, non-mitochondrial reads. The chromosome (chr) of origin and strand of a transcript (+) or (-) are indicated.

These very long intergenic RNAs were found at significantly greater abundance in cell lines derived from malignancies, such as K562, compared to RNA from the normal liver and brain (Figure 4c and 4d). In order to further investigate this in cancer, we analysed the six EFT samples and found many very long intergenic transcribed regions specific to these EFT samples, one example of which appears in Figure 5a. A region of approximately 650 kb on chromosome 7 was observed in total RNA from the EFT samples, but not in RNAs from K562, normal liver or the brain. Interestingly, transcription from this region occurs in at least three of the six EFT samples, including a cell line and two solid tumour samples, suggesting that it is not an artifact of *in vitro *cell culture. Furthermore, we detected long, intergenic transcribed regions specific to K562 cells that were transcribed from an approximately 300 kb intergenic region on chromosome 21 and not found in EFT samples (Figure 5b). While any given genomic region could be rearranged, amplified or deleted in the cancerous cells, it is unlikely that the same regions are rearranged in samples derived from different sources as exemplified by the EFT samples derived from four different patients.

In order to identify all such expressed intergenic regions, we pooled non-genic reads from the EFT samples and K562 cells and defined intergenic RNA domains of at least 50 kb in length (Materials and Methods section). Overall, 580 such domains were found (Additional File 4: Table S3). We thus propose to classify these intergenic regions as very long intergenic non-coding (vlinc) regions. As described below, expression from vlinc regions can be substantial and significantly higher in cancerous tissues than in normal tissues.

The total stable RNA output of a vlinc region can be quite substantial based on relative mass. In the case of K562, if both the vlinc and known protein-coding transcripts are ranked by relative mass, the top expressed vlinc (coordinates chr7:119013253-119337443, Additional File 4: Table S3) has a higher relative mass than the ninth most expressed known transcript which corresponds to the eukaryotic translation elongation factor 2, *EEF2 *(Additional File 5: Table S4). Of further interest is the observation that seven of the top eight expressed annotated transcripts in this cell line correspond to annotated nc-RNAs, such as *MALAT1 *and *RMRP *(Additional File 5: Table S4). Thus, the only protein-coding RNA that is higher in relative mass than the top vlinc RNAs is an isoform of epsilon globin gene (*HBE*).

Table 1 illustrates the tissue specificity of the K562 vlincs by comparing the ranks of the top 15 vlincs (ranked based on their relative mass) in this cell line to their ranks in other tissues both tumour and normal. As one can see, the ranks of the top 15 vlincs are significantly higher in other tissues. Also, Table 1 shows the ranks of the top 15 vlincs if they were ranked with the 65,260 UCSC Genes: the ranks would range from 9-100 among the ~65 K annotated genes again underscoring the fact that these regions are quite abundant.

**Table 1:** Ranking of the top 15 very long intergenic non-coding (vlincs) in the K562 cell line in other tissues [Ewing Family of Tumours (EFT) and normal) and among University of California Santa Cruz (UCSC) genes

ID	Chromosome	start	Stop	Relative mass (%)	K562 rank	Average rank EFT tumours	Minimum rank EFT tumours	Average rank liver and brain	Minimum rank liver and brain	Rank among UCSC genes
Vlinc_470	chr7	119013253	119337443	0.172	1	412	199	367	278	9

Vlinc_352	chr4	127557220	128001463	0.160	2	344	265	201	148	11

Vlinc_479	chr7	145112991	145362753	0.158	3	18	6	1	1	12

Vlinc_518	chr8	130323251	130656488	0.157	4	342	229	238	104	13

Vlinc_500	chr8	91302893	91655164	0.131	5	382	353	205	189	18

Vlinc_568	chrX	120153582	120594672	0.078	6	281	241	129	63	40

Vlinc_94	chr12	90597529	90798365	0.070	7	492	461	375	331	48

Vlinc_453	chr7	10409634	10632037	0.064	8	325	200	275	194	62

Vlinc_454	chr7	10638258	10786122	0.060	9	488	463	466	460	74

Vlinc_203	chr18	73753244	73834670	0.055	10	566	547	558	543	80

Vlinc_180	chr16	83939419	84202366	0.052	11	93	55	18	8	86

Vlinc_157	chr16	7897639	8105614	0.052	12	476	432	323	244	87

Vlinc_77	chr12	23219253	23449659	0.050	13	504	448	354	298	93

Vlinc_377	chr5	53642299	53764051	0.048	14	477	363	449	440	97

Vlinc_93	chr12	90280166	90554683	0.048	15	454	410	205	94	100

The total stable RNA output of vlinc regions tends to be higher in cancerous tissues than in normal ones. The maximum number of normalized counts for the seven tumour (6 EFT tumours and K562) and two normal samples (liver and brain) was calculated for each vlinc (Additional File 4: Table S3). The tumour/normal fold ratio of the maximal counts was then calculated for each vlinc (Additional File 4: Table S3). The median ratio was approximately 4.8, showing that most of the vlincs are more abundant in the tumour samples (*T*-test, *P*= 5.5 × 10^-27^). Furthermore, of the 580 vlinc regions, 427 had a ratio that was double or more and, for 222 vlincs, this ratio was 10 times higher. In contrast, there were three and zero vlinc regions whose maximal read count in a normal tissue was two and 10 times higher, respectively, than that in a tumour tissue/cell line.

Additionally, the total stable RNA output of vlinc regions can differentiate between different tumours and even tumours of the same category derived from different patients. A non-parametric Spearman correlation analysis among all of the pair-wise combinations of the EFT (six cell lines/tissues, two pairs from the same patient and one pair from a different patient), normal brain and liver sample and the K562 leukaemia cell line was performed based on the expression values for vlinc and UCSC Genes (Additional File 4: Table S3 and Additional File 5: Table S4). The following average correlation metrics based on all relevant pair-wise correlations were then calculated: between the EFT samples from the same patient; between the EFT samples from different patients; between the EFT and the normal tissues; and between the EFT samples and the leukaemia cell line. The correlation matrix is shown in Additional File 6: Table S5. For the UCSC Genes, sum of expression within each annotated transcript of the UCSC Genes database was calculated across all samples (Additional File 3: Table S2). The transcripts were then sorted by the sum expression level and the top two quartiles (Q1-Q2) were used to calculate the correlations. This was done to avoid inflation of the correlations due to transcripts not expressed in any of the tissues. The average correlation between the EFT samples from the same patient was, as expected, quite high using either the expression of UCSC Genes or vlincs (0.89 and 0.87, respectfully). It is worth noting that the pair of EFT samples from the same patient were represented by the primary and metastatic tumour isolated at significantly different time periods (months) from each other and, in the case of the one pair represented by the CHLA-9/-10 cell lines, cultured independently. This suggests that the expression of vlinc regions can be maintained for significant periods of time and suggests an important biological role. The average correlation between the EFT samples from the different patients was still high using expression of UCSC genes but lower using vlincs: 0.72 and 0.53, respectively. The latter was similar to those calculated between EFT samples and normal tissues: 0.36 and 0.49 based on UCSC genes and vlinc regions, respectively. Interestingly, expression levels of vlincs in EFT tumours were extremely different from the leukaemia K562 cell line (average correlation of 0.02). However, the average correlation between the two tumour types using known genes was still relatively high: 0.51. Although the number of tumour tissues used in this study is low, these observations suggest that the 'dark matter' RNAs produced from the vlinc regions can differentiate between different tumours, perhaps even better than the annotated genes. The ability to differentiate molecularly defined subgroups in the case of the EFT and, probably other tumours, is especially interesting since tumours are known to be heterogeneous and, in the case of EFT, are often referred to as a 'family of tumours'. Further, the diagnosis of EFT is often challenging due to its non-variant, microscopic appearance [25,26]. This data further confirms the notion of heterogeneity and suggests that the 'dark matter' RNAs could, in fact, be as good as or even better a discriminator than other methods, including coding RNAs, which are not unique to any given tumour, for identifying molecular subgroups within this family of tumours or to distinguish it from other tumour types.

Previously, human large intergenic non-coding (link) RNAs were identified in normal human embryonic and stem cell lines [27] and we sought to determine whether the link regions were overlapping. In fact, the majority of the link transcribed regions we have identified did not overlap the known human lacuna regions and, thus, represent novel RNAs that are also large, interagency and non-coding, as exemplified in the four examples shown (Figure 4c and 4d, Figure 5a and 5b). These latter regions have known lacuna regions located nearby, without overlap, while the former do not have lacuna regions in their vicinity. Furthermore, the interagency regions identified here achieve much greater lengths than known lacunas, with a median size of ~84 kb versus 21 kb for the lacunas (significant at *P *= 1.72 × 10^-53^, *t*-test). Overall, 37% (215/580) of the regions overlapped the K4-K36 domains harbouring lacunas as reported by Khalid *et al. *[27]. However, even when overlapping, the lacuna regions corresponded to only a fraction of our interagency regions: the percent of base pairs in the interagency regions found here only overlapped the lacuna regions by approximately 19% (13.51/68.51 Map). However, the overlap between the two categories of the interagency transcribed regions is highly significant (*P*-value < 10^-16^, chi-square test). This is interesting because the two regions were detected using different experimental approaches and even more so, they were found in very different types of tissues. Links were originally found in normal embryonic tissues/cell lines, while links were originally found in tumour tissues. This suggests a tantalizing possibility that links are also expressed very early in development, perhaps at very specific stages of development, which explains why they so far have eluded detection and the determination of function during early development.

We also analysed whether vlincs could be conserved across species, at least in terms of the syntenic location. In order to do this we took advantage of the data denoting 5' and 3' ends of mouse transcripts (the reliable cluster dataset) from the FANTOM3 consortium [5]. We took the coordinates of 181,047 such clusters and converted these clusters into regions of the hg18 version of the human genome (Materials and Methods section). Overall, 165,169 clusters could be remapped to hg18. Of the 165,169 clusters, 7456 were intergenic and, of those, 172 spanned at least 50 kb of genomic space - the minimal size of a vlinc region. These 172 clusters are thus analogous to our vlinc regions based on size and location in the intergenic regions. Overall, 15 vlinc regions were found to overlap 21 (out of 172) FANTOM3 clusters by at least 10 kb (Additional File 7: Table S6). Sometimes a vlinc region overlapped more than one cluster (Additional File 7: Table S6). Overall, given the small span of the genomic space covered by the vlincs and the 172 FANTOM3 clusters, the overlap was significant (*P*-value < 10^-16^, chi-square test). Furthermore, in the cases of 13 vlincs, the region of overlap was more the 50% of the length of either the vlinc or the FANTOM3 cluster it overlapped (Additional File 7: Table S6).

Thus, some vlincs are conserved in mouse at least in the syntenic locations. The fact that only 15 (or 13 if more stringent criteria above are used) vlincs are conserved in mouse is probably due to the fact that: (1) only 172 such regions were identified in FANTOM3, probably due to usage of polyA+ RNA for library constructions by the FANTOM3 consortium, significantly lowering potential for overlap; and (2) differences in biological material - vlincs appear to be highly tissue specific.

It is difficult to currently estimate the complexity and properties of RNA molecules harboured within the vlinc regions. What is currently annotated explains a minute fraction of the complexity found in these regions, both in terms of the base pair coverage and fraction of reads, even if we include additional databases, such as RefSeq, Ensemble and all spliced ESTs. For example, exons of the spliced ESTs cover only ~0.6% of the genomic sequences covered by all vlincs: 434,530 out 68,509,810 bp correspondingly. Also, taking K562 as an example, approximately 2% of all reads (7,745/358,600) that fall within the vlinc regions can be accounted for by the exons of the spliced ESTs. This number climbs to 3.5% if exons of all spliced ESTs, UCSC Genes, RefSeq and Ensemble are combined (12,392/358,600 reads). As mentioned above, however, when summarized over each vlinc region, these transcripts are quite abundant based on how many reads one needs to minimally cover all non-ribosomal, non-mt RNAs in a human cell. Nevertheless, elucidation of the structures of transcripts within the vlinc regions is of paramount importance. However, this can only be done by targeting each region and cloning and sequencing each full-length cDNA, to the extent that this is even possible. Both cloning and 5' and 3' RACE (Rapid Amplification of cDNA ends) experiments may prove impossible if the individual transcripts turn out to be very long, necessitating new strategies for characterizing these regions.

Given that we sequenced total as well as polyA-selected RNA, we can also estimate the relative mass of non-ribosomal, non-mt RNAs in both fractions and also estimate the number of reads required to fully sequence this fraction from one cell. Sequencing of total RNA not depleted of ribosomal RNA yields a value of about 6% (range 4%-8%) of non-ribosomal and non-mt RNA as a fraction of total RNA in cell by mass. Based on the amount of RNA present in one mammalian cell (~20 pg) and an average read of 35 bases (representing 2 × 10^-20 ^g RNA), we would need greater than 100 million reads to sequence all non-ribosomal, non-mt RNA in a single human cell. Since the number of reads in this study of RiboMinus RNAs represents only a fraction of the total number of reads estimated above, we can safely assume that we sequenced less than one cellular equivalent of RNA. Thus, the readily apparent detection of the above non-exonic RNAs, as shown in Figures 3-5, suggests that their expression occurs at significant levels compared to protein encoding RNAs and certainly much higher than one copy per cell. We can also estimate that the polyA-selected material probably represents only a small fraction (approximately 5%-25%) of non-ribosomal, non-mtRNA. This estimate follows from the typical yield of a single polyA selection, which represents approximately 1%-2% of starting total RNA amounts, of which approximately 50% is still ribosomal or mtRNA and, from the observation made in this study, that total RNA contains on the order of 6% (range 4%-8%) non-ribosomal and non-mt RNA.

## Conclusions

In summary, we show that the relative mass of 'dark-matter' RNAs is both substantial and, in some cases, greater than that of protein encoding transcripts. We find that the 'dark-matter' RNA fraction typically represents 50%-65% of all non-ribosomal, non-mt RNA by mass in many normal and neoplastic tissues. We derived these estimates using single-molecule sequencing of cDNAs that avoids amplification, ligation and cDNA size-selection steps, all of which are known to increase the potential for bias prior to sequencing [28,29]. This number is probably an underestimate since we considered only reads that fall within non-exonic regions. Exonic sequences that are part of transcripts that have no known function were not counted due to the limitations discussed earlier. Also, because the issues with reverse-transcriptase mentioned above make it difficult to unambiguously define the strand of all RNAs, antisense exonic reads would be counted as exonic reads, also decreasing the fraction of the true 'dark matter' RNAs. The minimal sample preparation employed in this study more closely reflects the native RNA population abundance compared with amplification-based, next-generation platforms. We also find that total cellular RNA has a much higher complexity than polyA+ RNA, which is highly enriched for transcripts comprised of currently annotated exonic sequences across a variety of cell types and tissues. A methodological choice that assumes RNAs need to be polyadenylated in order to be worthy of study by discarding everything without a polyA tail is, thus, certain to leave gaping holes in understanding of the transcriptome. We further show that the intergenic space in regions devoid of known, annotated transcripts harbours very long stretches (for example, hundreds of kb), of abundantly transcribed and likely non-protein encoding RNA species, even though we cannot exclude a possibility that some of transcripts in the vlinc regions may encode proteins. Our understanding of the repertoire of these types of RNAs remains far from complete, as we have identified hundreds of new loci by profiling just seven samples from two types of tumours. Furthermore, the statistically significant overlap between the intergenic vlinc RNAs found in tumours in this work and the lincRNAs found in the normal human embryonal and stem cells [27] raises the interesting hypothesis that these RNAs in the vlinc regions function in specific capacities early in development, only to be silenced in differentiated cells and reactivated during malignant transformation. These transcripts appear to be readily identifiable in tumours, particularly poorly differentiated, 'stem cell like' tumours such as EFT, but less so in normal tissues, which suggests functional roles in both development and oncogenesis. Such expressed RNA species may also have potential usefulness as diagnostic biomarkers for specific cancer types, as exemplified previously by the *HOTAIR *lincRNA [30] and other non-coding RNAs [31] as well as in other diseases [32]. It would be interesting to determine whether vlincs share common functional properties with macro ncRNA identified as regulators of imprinting [33]. Further analysis of more tumours from a variety of individuals will be required in order to establish conclusive connections between expressed regions and cancer aetiology.

## Materials and methods

### RNA

Total RNA was obtained from commercial sources for the K562 cell line (Ambion, Texas, USA) and normal liver and normal brain tissues (Clontech, CA, USA). Total RNA was isolated from a pair of cell lines from a patient with EFT, one established at diagnosis (CHLA-9) and the second from a nodal metastasis obtained after chemotherapy (CHLA-10) [34]. Most EFT tumours express a fusion protein (EWSR1/FL1-1 or equivalent) generated from a translocation and fusion between *EWSR1 *and an ETS family gene. The fusion protein acts as a transcription regulator and transcriptome sequencing of this tumour is probably to reveal mis-expressed RNAs involved in neoplastic transformation. Cell lines tested mycoplasma-free. Cell line identity was validated using patient bone marrow by the short tandem repeat (STR) assay [35] and verified by STR at time of nucleic acid extraction for these experiments. Cell lines can be obtained from the Children's Oncology Group repository at http://www.COGcell.org. The EFT tissues were obtained from the repository at the Children's Hospital of Los Angeles, USA, and the detail of the specimens could be obtained from Dr Timothy Triche [triche@usc.edu]. Briefly, their properties were as follows:

1. Primary No.2 tumour: sacral tumour; age -13 years and 9 months; EWSR1 gene rearrangement positive by FISH

2. Metastasis No.2 tumour: lung metastasis; same patient as Primary No.2 tumour

3. Primary No.3 tumour: paraspinal tumour; age - 16 years and 9 months; *EWSR1 *gene rearrangement positive by FISH

4. Metastasis No.4 tumour: metastasis of a primary thoraco-abdominal tumour; age -14 years and 8 months; real-time polymerase chain reaction and sequence analysis confirmed type I fusion of *EWSR1/FLI-1 *genes.

Before further fractionation, total RNA was treated with DNase I as follows: 50 μg of total RNA was mixed with 10 μL of 10X DNase I buffer (Roche); 2 μL of RNaseOut (Invitrogen, CA, USA); and 8 μL of recombinant DNase I (10 U/μL, Roche, CA, USA) and incubated for 45 min at 37°C. The RNA was then purified using the RNeasy MinElute kit (Invitrogen).

The DNase I-treated total RNA was either unfractionated (total RNA) or fractionated using one of the following methods: (1) depleted of ribosomal RNA (rRNA) using the RiboMinus kit (Invitrogen); (2) polyA+ fraction was selected using a magnetic bead-based purification kit (Dynabeads mRNA purification kit, Invitrogen); or, (3) polyA+ fraction was selected using the oligo-dT cellulose method (Micro Poly(A)Purist Kit, Ambion, Texas, USA).

### Preparation of RNA for sequencing

Except where noted, 100-400 ng of DNase I -treated RNA was mixed with the following reagents from the SuperScript III kit (Invitrogen). First, 10 μL of 50 ng/μL Random Hexamers and 2 μL of 10 mM dNTPs were added in the total volume of 25 μL. The mixture was then placed in a thermocycler and heat denatured at 65°C for 5 min followed by rapid cooling on ice. Next, 5 μL of 10X cDNA synthesis buffer, 5 μL of 0.1 M DTT and 10 μL of 25 mM MgCl_2 _were added. The samples were returned to the thermocycler and allowed to incubate at 15°C for 20 min. Then, 2.5 μL of RNaseOut and 2.5 μL of SuperScript III reverse transcriptase were added and the samples incubated at 25°C for 10 min, 42°C for 40 min, 55°C for 50 min and 70°C for 10 min.

After reverse transcription, RNA was removed by adding 1 μL of RNaseH (Invitrogen) and 1 μL of RNase If (New England BioLabs, MA, USA) to each sample and incubating at 37°C for 30 min. The cDNA was then purified by two rounds of purification over Performa columns (EdgeBio, MD, USA) and quantified using a NanoDrop spectrophotometer.

Next, a 3' poly-A tail was added to the cDNA samples. cDNA (100 ng) was mixed with a control oligo to monitor tail length and water in a total volume of 33.5 μL. The mixture was denatured at 95°C for 5 min followed by rapid cooling on ice. Five microlitres of 2.5 mM CoCl_2_, 5 μL of 10x terminal deoxynucleotidyl transferase (TdT) buffer (New England BioLabs), 5 μL of 50 μM dATP and 1.5 μL of TdT (20 U/μL, New England BioLabs) was then added and the samples incubated at 42°C for 1 h and at 70°C for 10 min.

The 3' ends of the poly-A-tailed cDNA were then blocked with biotin-ddATP. The sample was denatured at 95°C for 5 min followed by rapid cooling on ice. We then added 0.3 μL of 1 mM biotin-ddATP (Perkin Elmer, CA, USA) and 1.5 μL of TdT followed by incubation at 37°C for 45 min and 70°C for 10 min.

The control oligo was removed by digestion with the USER enzyme (New England BioLabs). Then 1 μL of the USER enzyme (1 U) was added to the sample and incubated at 37°C for 30 min.

The sample was then purified using AMPure beads (Agencourt, MA, USA) by bringing the volume up to 60 μL with water and adding 72 μL of the AMPure beads followed by incubation at room temperature for 30 min with agitation. The beads were then captured on a magnetic stand and washed twice with 70% ethanol. The beads were allowed to air dry for 5-7 min, resuspended in 20 μL of water and left open for 30 min on the magnet. The eluate was collected and the beads were resuspended again in 20 μL of water and then left for 5 min on the magnet. The eluate was collected again and combined with the first eluate.

Typically, samples were hybridized to the HeliScope flow cell in 20 μL at a loading concentration of 100-350 pM.

### SMS reads to the genome

SMS reads were trimmed for leading T homopolymers and were filtered for reads with a minimal length of 25 bases after trimming using a suite of Helicos tools available at http://open.helicosbio.com/mwiki/index.php/Releases and described at http://open.helicosbio.com/helisphere_user_guide/index.html. Alignments were conducted with indexDPgenomic software freely available on the Helicos website http://open.helicosbio.com/mwiki/index.php/Releases. The aligner maximizes the aligned yield of SMS reads due to the ability to align reads in which the predominant error is represented by deletions common in single-molecule sequencing. For the genomic alignments, reads were aligned to the NCBIv36 version of the genome supplemented with the complete ribosomal repeat unit (Gen Bank Accession U13369.1) or the DM3 version of the fly genome supplemented with the complete ribosomal repeat unit (Gen Bank Accession M21017.1) using the Helicos BASIC analysis pipeline http://open.helicosbio.com/helisphere_user_guide/index.html. The sequence reads were filtered to include reads with a minimal length of 25 bases and aligned using a stringent normalized score of 4.5 (human genome) and 4.3 (fly genome). Aligned reads were further filtered for reads having a unique best alignment score. The mapped reads were then compared to UCSC genes and FlyBase gene annotations to partition them into exonic, intronic and intergenic reads.

The normalized score was defined as follows:

Score = (No. matches*5 - No. mismatches*4)/length_read

For example, in the following alignment:

Tag Sequence CCTCCGTGTTGTTCCAGCC-CAGTGCTCGCAGG

Ref Sequence C-TCCGTGTTGTTCCAGCCACAGTGCTCGCAGG

Length of alignment block: 33

Length of tag sequence: 32

Number of matches: 31

Number of errors: 2

Score: (31*5) - (2*4) = 155 - 8 = 147

Normalized score = 147/32 = 4.59375

### Identification of the vlinc RNA domains

Reads not overlapping the genic regions from the Ewing tumours and K562 were pooled and used to generate the densities of read coverage throughout the genome. The vlinc domains were defined in the Integrated Genome Browser http://www.bioviz.org/igb/download.shtml by applying the following thresholds to the density graphs for each chromosome:

Threshold = 80th percentile

MaxGap = 5000

MinRun = 50000

### Comparison with the FANTOM 3 dataset

The coordinates of the 181,047 reliable 5'/3' clusters described in the Table 3 of the FANTOM3 consortium paper [5] were downloaded from the FANTOM3 website at ftp://fantom.gsc.riken.jp/FANTOM3/boundary_set/pair53_clusters.txt.gz. The coordinates were lifted from the mm5 version of the mouse genome to hg18 in a two step process using the UCSC liftOver tool: mm5- > mm8 and mm8- > hg18.

## Abbreviations

EFT: Ewing Family of Tumours; FISH: fluorescent *in situ *hybridization; lincNAs: large intergenic non-coding; mtRNA: mitochondrial RNA; ncRNA: non-coding RNA; rDNA: recomninant DNA; vlinc: very long intergenic non-coding; SMS: single molecule sequencing; STR: short tandem repeat; TdT: terminal deoxynucleotidyl transferase.

## Authors' contributions

PK, PM, JFT and TJT designed the study. GsL, CPR and TJT provided the experimental material. PK performed the analysis. PK, GsL, RJA, JFT and TJT wrote the manuscript and provided input on the analysis and the presentation of the data. PK, TR and FO performed the molecular biological experiments. PHBS and GR provided intellectual contribution to the manuscript. All authors read and approved the final manuscript.

## Supplementary Material

Additional File 1 **Definition of the 'dark matter' RNA**. Provides a definition of what kind of RNA molecules should be included in the 'dark matter' RNA realm and argumentation for their inclusion. Click here for file

Additional File 2 **Table S1**. Distribution of single-molecule sequencing reads obtained from polyA+, rRNA-depleted (Ribominus) and total RNA from different tissues among different types of annotations. Click here for file

Additional File 3 **Table S2**. Distribution of single-molecule sequencing reads obtained from ribosomal RNA-depleted (Ribominus) RNA from Ewing Family of Tumours solid tumours and cell lines. Click here for file

Additional File 4 **Table S3**. Coordinates of the very long intergenic regions and their normalized expression levels in each tissue. Click here for file

Additional File 5 **Table S4**. Normalized expression levels of University of California Santa Cruz genes in each tissue. The total number of reads within each of the regions was normalized to 10 M non-ribosomal non-mitochondrial unique reads in each sample. Click here for file

Additional File 6 **Table S5**. Spearman correlation matrix based on expression of very long intergenic non-codings and University of California Santa Cruz genes between different tumour and normal tissues Click here for file

Additional File 7 **Table S6**. Overlap between very long intergenic non-codings and FANTOM3 intergenic clusters of over 50 kb. Click here for file
