# The majority of total nuclear-encoded non-ribosomal RNA in a human cell is 'dark matter' un-annotated RNA

**DOI:** 10.1186/1741-7007-9-86

**Published:** 2011-12-20

**Authors:** Philipp Kapranov, Georges St Laurent, Tal Raz, Fatih Ozsolak, C Patrick Reynolds, Poul HB Sorensen, Gregory Reaman, Patrice Milos, Robert J Arceci, John F Thompson, Timothy J Triche

**Affiliations:** 1 Helicos BioSciences Corporation, One Kendall Square, Building 700, Cambridge, MA 02139, USA; 2 Department of Molecular Biology, Cell Biology and Biochemistry, Brown University, SFH Life Sciences Building, 185 Meeting St, Providence, RI 02912, USA; 3 Cancer Center, Departments of Cell Biology & Biochemistry, Pediatrics, and Internal Medicine, School of Medicine, Texas Tech University Health Sciences Center, 3601 4th Street STOP 9445, Lubbock, TX 79430-6450, USA; 4 British Columbia Cancer Research Centre, 675 West 10th Avenue, Room 4112, Vancouver, BC, Canada V5Z 1L3; 5 Department of Pediatrics, The George Washington University School of Medicine and Health Sciences, Division of Oncology, Children's National Medical Center, 11 Michigan Ave, NW, Washington, DC, 20422, USA; 6 Kimmel Comprehensive Cancer Center at John Hopkins, Department of Oncology/Pediatric Oncology, The Buntings Blaustein Cancer Research Building, 1650 Orleans Street, Suite 207, Baltimore, MD, 21287, USA; 7 Department of Pathology, University of Southern California, 1975 Zonal Avenue, Los Angeles, CA 90089-9034, USA; 8 Grupo de Inmunovirologia, SIU, Universidad de Antioquia, Calle 67 Número 53 - 108, Medellin, Antioquia, Colombia

## 

The publisher notes typographical errors which were introduced to the first paragraph of page 10 (beginning "Previously, human large intergenic non-coding (linc) RNAs were identified in normal human embryonic and stem cell lines...") during the production process. The publisher apologises for the errors caused. The online article HTML was corrected on 19 October 2011, and the article PDF on 31 October 2011; readers with previous versions of the article are advised to update to the corrected version [1].

Corrected paragraph:

"Previously, human large intergenic non-coding (linc) RNAs were identified in normal human embryonic and stem cell lines [twenty seven] and we sought to determine whether the vlinc regions were overlapping those. In fact, the majority of the vlinc transcribed regions we have identified did not overlap the known human lincRNA regions and, thus, represent novel RNAs that are also large, intergenic and non-coding, as exemplified in the four examples shown (Figure four c and four d, Figure five a and five b). These latter regions have known lincRNA regions located nearby, without overlap, while the former do not have lincRNA regions in their vicinity. Furthermore, the intergenic regions identified here achieve much greater lengths than known lincRNAs, with a median size of ~84 kb versus 21 kb for the lincRNAs (significant at P = 1.72 × 10^-53^, t-test). Overall, 37% (215/580) of the vlinc regions overlapped the K4-K36 domains harbouring lincRNAs as reported by Khalil et al. [twenty seven]. However, even when overlapping, the lincRNA regions corresponded to only a fraction of our intergenic regions: the overlap of base pairs in the intergenic regions found here with the lincRNA regions was only approximately 19% (13.51/68.51 Mbp). However, the overlap between the two categories of the intergenic transcribed regions is highly significant (P-value < 10^-16^, chi-square test)."
