# Supplementary material for: The majority of total nuclear-encoded non-ribosomal RNA in a human cell is 'dark matter' un-annotated RNA
Source: BMC Biol. 2010 Dec 21;8:149. doi: 10.1186/1741-7007-8-149 (PMC3022773; doi:10.1186/1741-7007-8-149)
Supplement: Additional File 1 — Definition of the 'dark matter' RNA. Provides a definition of what kind of RNA molecules should be included in the 'dark matter' RNA realm and argumentation for their inclusion. [file 1741-7007-8-149-S1.pdf]

Definition of “dark matter” RNA.

The term “dark matter transcription” has not been rigorously defined despite the controversy over its importance. To help clarify the discussion, we define “dark matter” RNAs as any transcripts whose function we do not understand similar to the dark matter of the universe which we still do not understand. These RNAs may have function or they may be just by-products or errors in cellular processes such as splicing or inappropriate transcription; all we know is that they do exist.

Any RNA which is annotated as coding for a protein is considered of known function even if the function of the protein is not known. Any non-coding RNAs with known functions (i. e. tRNAs, rRNAs, miRNAs, etc) would not be dark matter. We propose any transcript that fulfills either of the following two criteria should be considered as a “dark matter transcript”: 1. Its complete sequence might not be known but its existence is known and it is different from the sequence of transcripts whose function is known. The evidence of the latter could be based either on the knowledge of its partial sequence, such as provided by a short read from a next-generation sequencing technology or EST data base or, indirectly, through hybridization to probes on a microarray. 2. Its complete sequence is known, but its function is not.

This definition leads to following immediate key corollaries: 1. Transcripts in category 1 may or may not be protein-coding; the key point is it they are presently unknown or un-annotated and thus fall into the “dark matter” category. 2. Transcripts in the category 1 or 2 may be outside the bounds of a locus of a known function or may overlap it. It may even borrow sequences (e.g., exons of protein-coding messages) that are otherwise parts of different transcripts of known function or it may even be mostly composed of such sequences. For example, the results of the GENCODE annotations suggested that for 487 compiled human loci in ENCODE pilot regions there are 2,608 transcripts, of which 1,097 are coding [36]. The remaining 1511 non-coding RNAs would also fall into the “dark matter” category. 3. An annotated transcript, for example, in UCSC Genes or Ensemble, can be a “dark matter” transcript if its function is not understood. For example, an annotation without any obvious

protein-coding capacity and also not having a known function as a non-coding RNA, is a “dark matter” RNA.

Due to the limitation imposed by length of a sequence read in SMS and most other next-generation sequencing technologies, the entire sequence of a transcript represented by a ‘read’ cannot be known. Thus, we can not measure the “dark matter” RNAs in category 2 above. We can only measure the fraction of RNAs in category 1 and thus our estimate of the total “dark matter” RNA will be an underestimate. Furthermore, since “dark matter” RNAs can be both inside and outside of the bounds of annotated transcripts, we assign reads both in intronic and intergenic regions to the “dark matter” RNAs as long as these reads do not overlap annotated exons defined by UCSC Genes.

While we can not be sure how much of the un-annotated transcription belongs to the non-coding (ncRNA) class, it is not unreasonable to assume that most of it is. This assumption is based on the fact whenever systematic analysis of the sequenced cDNAs was undertaken, for example by the FANTOM consortium [5, 37, 38] or by the GENCODE annotation group [36], the majority of RNAs that did not correspond to annotated protein-coding messages also did not seem to encode proteins. This holds true even for transcripts that share exons with protein-coding RNAs. For example, the results of the GENCODE annotations suggested presence of on average 5.4 isoforms per human protein-coding locus with only 2.3 isoforms having coding potential [36]. Thus, it is not unreasonable to assume that the majority of RNA detected by us are non-coding.

To some extent, this definition of “dark matter” is problematic because the fraction of RNA falling into this category is constantly changing as function becomes assigned. Indeed, driving this fraction of what is considered dark matter to zero and achieving a full understanding of what role all RNAs play is exactly what is hoped to be achieved over time.
